# Recognizing the Psychosocial Aspects of Type 1 Diabetes in Adolescents

**DOI:** 10.4274/jcrpe.1745

**Published:** 2015-03-05

**Authors:** Erdal Adal, Zerrin Önal, Atilla Ersen, Koray Yalçın, Hasan Önal, Ahmet Aydın

**Affiliations:** 1 Medipol University Faculty of Medicine, Department of Pediatric Endocrinology and Metabolism, İstanbul, Turkey; 2 Kanuni Sultan Süleyman Training and Research Hospital, Clinic of Pediatrics, İstanbul, Turkey; 3 Kasımpaşa Military Hospital, Clinic of Pediatrics, İstanbul, Turkey; 4 Okmeydanı Education and Research Hospital, Clinic of Pediatrics, İstanbul, Turkey; 5 Kanuni Sultan Süleyman Training and Research Hospital, Clinic of Pediatric Endocrionology and Metabolism, İstanbul, Turkey; 6 İstanbul University Cerrahpaşa Faculty of Medicine, Department of Pediatric Metabolism Diseases, İstanbul, Turkey

**Keywords:** type 1 diabetes, adolescent, depression, anxiety, social support

## Abstract

**Objective:**

Considering the ever increasing population of diabetic adolescents and the association of the disease with psychosocial problems throughout its course, depression and/or anxiety and social support from parents are issues of special concern in these patients. The study aimed to identify the depression and anxiety state of diabetic adolescents and its impact on the management of diabetes mellitus (DM).

**Methods:**

295 adolescents with type 1 DM and their parents attended our study. Psychological distress was assessed using the Children’s Depression Inventory and the State-Trait Anxiety Inventory (STAI I-II) for Children, Perceived Social Support from Family (PSS-Fa) scale, Beck Depression Inventory for adults, STAI I-II for adults and the Multidimensional Scale of Perceived Social Support (MSPSS). Records of glycemic measurements, insulin dosage and hemoglobin A1c levels were used as glycemic control parameters.

**Results:**

Depression rate was 12.9%. State (p<0.001) and trait anxiety (p<0.001) levels were high; PSS-Fa (p<0.001) and MSPSS (p<0.006) scores were low in the depressive patients. Positive correlations were noted between depression, PSS-Fa, STAI-I and STAI-II.

**Conclusion:**

Therapeutic strategies of DM should include co-existing psychiatric conditions throughout the course of the disease. In diabetic adolescents, PSS-Fa, STAI-I and STAI-II appear to be effective tools in the evaluation of depression.

## INTRODUCTION

The period of adolescence is of special concern for parents and caretakers due to the changes occurring in the individual’s biological and intellectual make-up and also in his/her relationship to his/her socio-economic environment. Difficulties in adjusting to these changes may lead to emotional distress and may be manifested as depression or anxiety. Presence of a chronic disease may have an important influence on the physical, psychological and emotional state of the adolescent and can limit his/her ability to cope with the necessary tasks of everyday life. Thus, an adolescent who has a chronic illness needs to adapt to the changes in his/her biological and intellectual make-up on one hand and at the same time should be able to cope with the treatment of his/her condition and also with the stress caused by the presence of a chronic disease. Therefore, compatibility is difficult for adolescents and may expose them to many risks such as depression, anxiety and other psychological states (1).

Given the worldwide rise in diabetes mellitus (DM) as a chronic disease in recent years, an increase which will probably continue in the coming decades and the increased risk of poor diabetes outcomes associated with co-morbid depression especially among adolescents, DM care should take into consideration not only the endocrinological and physical outcomes of the condition, but also its psychiatric features (2). This study was designed to investigate the psychiatric features of type 1 DM in a group of Turkish adolescent diabetics.

## METHODS

This cross-sectional study aiming to explore the presence and extent of depressive symptoms in adolescents followed with a diagnosis of type 1 DM was carried out in the Pediatric Unit at the Ministry of Health Okmeydanı Research and Training Hospital and in the Pediatric Endocrine Unit at Kanuni Sultan Süleyman Research and Training Hospital in İstanbul, Turkey between the years 2006-2008. The study population consisted of 295 adolescents aged between 11 and 18 years and their parents. Patients with mental retardation as a possible obstacle to communication and those with co-morbid chronic disease were excluded. The study protocol was approved by the Ethics and Research Committee of the hospital and written informed consent was obtained from every parent.

Depressive symptoms in youth were assessed using the Children’s Depression Inventory (CDI), which is a self-report questionnaire consisting of 27 items (3). The CDI has wide use across chronic health conditions, specifically diabetes. Kovacs et al (4) has developed the inventory and the questionnaire has been modified for our population by Oy (5). A score of 19 is indicative of presence of significant depressive symptoms.

The State-Trait Anxiety Inventory (STAI-I, STAI-II) was used for assessment of patients’ stress levels. STAI-I measures the reactions of anxiety in a certain time and condition, STAI-II measures the permanence of anxiety independent of the circumstances (6). The inventory has been standardized for the Turkish population by Özusta (7).

To assess the level of “Perceived Social Support from Family” (PSS-Fa), we used the PSS-Fa scale developed by Procidano and Heller (8) and modified by Eskin (9) and Yıldırım (10) as a reliable method for the Turkish population; the scale is composed of 20 items.

Depression in parents who cared for type 1 DM adolescents was assessed using the Beck Depression Inventory (BDI) for adults. This self-report questionnaire consists of 21 items and is indicative of presence of significant depressive symptoms over a score of 17 (11,12).

Stress levels of individuals responsible for the care of type 1 DM patients were assessed using the STAI-II for adults. The validity and reliability tests of the inventory for the Turkish population were done by Oner and Le Compte (13).

The scale used to measure social support in this research was developed by Zimmet et al (14). This Multidimensional Scale of Perceived Social Support (MSPSS) consists of 12 items which measure three issues: 1) support perceived by the family members (four items), 2) support perceived by important persons (four items) and 3) support perceived by friends (four items). Based on 7-point Likert scale, all items of this scale ranged from ‘very strongly agree’ to ‘very strongly disagree’. The total score ranged from 12 to 84. MSPSS was adapted to the Turkish literature by Eker et al (15).

Number of daily serum glucose measurements, insulin dose per weight (insulin dose/kg) and hemoglobin A1c (HbA1c) levels were used as glycemic control parameters. The records of blood glucose measurement device were used to identify the number of daily serum glucose measurements.

### Statistical Analysis

Data analysis was performed using SPSS version 16.0. Student’s t-test was used to compare the relationship between quantitative variables, Spearman’s correlation analysis was used for data correlations and logistic regression was used for risk factor analysis. Confidence coefficient of the study was 0.95 and a significance level of p=0.05 was used for the statistical analysis.

## RESULTS

The study group consisted of 295 patients ranging in age from 11 to 18 years (mean age 14.7±1.9 years, females 57.6%) and their parents. Duration of type 1 DM was 5.93±3.71 years and mean HbA1c level was 9.58±2.1%. The education of the parents was primary school level in 80% (n=236) and higher in 20%. 92.8% (n=272) of the parents were married. Most of the parents who attended the study were mothers (89.5%).

CDI score was high in 38 patients (12.9%), indicating a state of depression. Mean age and number of blood glucose measurements showed significant differences between the depressive and non-depressive patients. Gender, diabetes duration, HbA1c levels and number of daily injections demonstrated no differences between the two groups. Particularly, state anxiety (p<0.001) and trait anxiety (p<0.001) levels were higher and level of PSS-Fa was lower (p<0.001) in depressive type 1 DM adolescents in comparison with the non-depressive ones. Depression, anxiety scales, marriage and graduate status of parents who cared for depressive type 1 DM adolescents were comparable with the status of parents of non-depressive adolescents, but MSPSS was statistically lower among the depressive individuals (p=0.006) (Table 1).

A low correlation was noted between depression, age and HbA1c level and a positive correlation was noted between PSS-Fa, STAI-I and STAI-II in correlation analysis (Table 2).

In the evaluation of factors that lead to depression, age and HbA1c level did not stand out as important risk factors in logistic regression analysis, but PSS-Fa, STAI-I and STAI-II scores appeared to influence the progression of depression. Low PSS-Fa score had a negative effect on depression (Table 3).

## DISCUSSION

Many diabetic adolescents have difficulty in undertaking the tasks they need to undertake to meet the long-term treatment requirements of their chronic condition. In previous studies, it has been reported that the number of daily glucose controls decreased and HbA1c levels increased during the adolescent period (6). Possibly, this situation is related to psychosocial problems encountered in the adolescent period and also to lack of sufficient social and psychological support from the family during this troublesome period. For these reasons, the patient’s state of anxiety linked to the chronic condition needs to be taken into consideration by all individuals involved in the care of these patients.

Kovacs et al (4) in a long-term controlled prospective study found that 42.4% of type 1 DM adolescents had a psychiatric disorder and depression was the most frequent type of disorder occurring in a ratio of 27.5%. In a study by Kokkonen et al (16), the prevalence of depressive symptoms which was 12% in the 8-12 years old diabetic group increased to 18% among adolescent diabetic patients. Jaser et al (11) reported the ratio of depressive symptoms as 12.3% among these individuals. Our results were compatible with these previous studies since we found a similar rate of 12.9% for depression in our type 1 diabetic patient group.

Some investigators, speculating on a possible gender effect on development of psychological disorders in diabetic adolescents, reported a higher incidence in females, but this finding was not confirmed by others (17,18,19). We also did not find any relationship between gender and depression in our study.

Some researchers suggested that a positive correlation could exist between higher HbA1c levels and depression among diabetic adolescents, but this finding is still on debate (20,21,22). In our study, HbA1c levels demonstrated a weak correlation with depression scale scores in our patients, nevertheless, it did not stand out as a risk factor for depression in the logistic regression analysis.

Whittemore et al (21) did not find any correlations of depressive symptoms with age and duration of disease in their study and reported the highest prevalence of depressive symptoms in diabetic adolescents who had a disease duration longer than 10 years. On the other hand, duration of disease was not found to be associated with psychosocial or depressive symptoms in most of the cross-sectional analyses (23,24). Actually, a weak correlation was found between age and CDI score in our study, but it was not a risk factor for depression in the logistic regression analysis. Moreover, in accordance with previous reports, duration of DM was not associated with depression. However, this finding may be related to the fact that our study group consisted of patients whose duration of disease was under 10 years.

A negative correlation was identified between number of daily blood glucose measurements and depression (20,22). Our findings did not confirm such a relationship. We found that the number of glucose measurements correlated with daily injections and HbA1c levels but not with CDI in our study group.

Herzer et al (25) reported a correlation between depression and anxiety in diabetic children and adolescents. Actually, a state of anxiety and its effect on the disease process have been mentioned in most of the international studies; however, this subject was underestimated in Turkey (7,8,9). We found a correlation between CDI and STAI I-II scores of the adolescents (Table 2). Furthermore, the patients’ state of anxiety was a significant risk factor for depression (Table 3).

Family-based psychosocial factors such as relationships within the family, emotional conditions and diabetes-specific support shown by the family correlate well with the psychosocial adaptation of diabetic adolescents (26,27,28). Expressiveness, cohesiveness, teamwork and diabetes-related family conflict are some potential values of a parent-adolescent partnership in diabetes management (29,30,31,32,33). Assessment of PSS-Fa showed us a negative correlation with anxiety and especially with depression; PSS-Fa was a significant risk factor for depression (Tables 2 and 3). Moreover, MSPSS analysis demonstrated a weak negative correlation with STAI-I, a weak positive correlation with the level of PSS-Fa, but no significant correlation with the depression scores of the patients. These last results showed us that parents who had social support may have a positive influence on adolescent diabetic patients, although social support of parents seemed to have no effect on depression in the diabetic adolescent but appeared to be effective on the anxiety status.

Considering the ever increasing population of diabetic adolescents in our country and the significant relationship of DM with psychosocial problems such as depression and/or anxiety throughout its course, the provision of psychological support and social support from their parents is an issue of special concern. Therapeutic strategies of DM should also consider the treatment of co-existing psychiatric conditions throughout the course of the disease.

## Figures and Tables

**Table 1:**
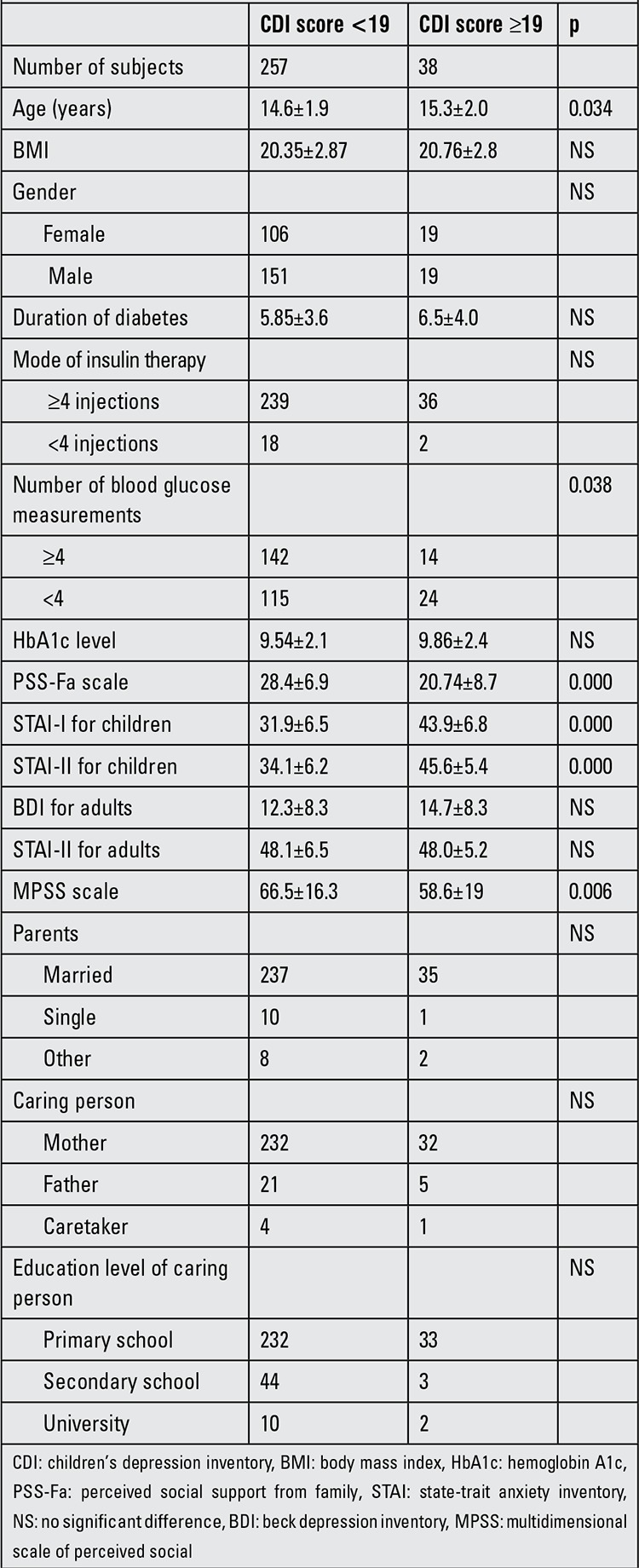
Data according to Children’s Depression Inventory (CDI) score in type 1 diabetic adolescents

**Table 2:**
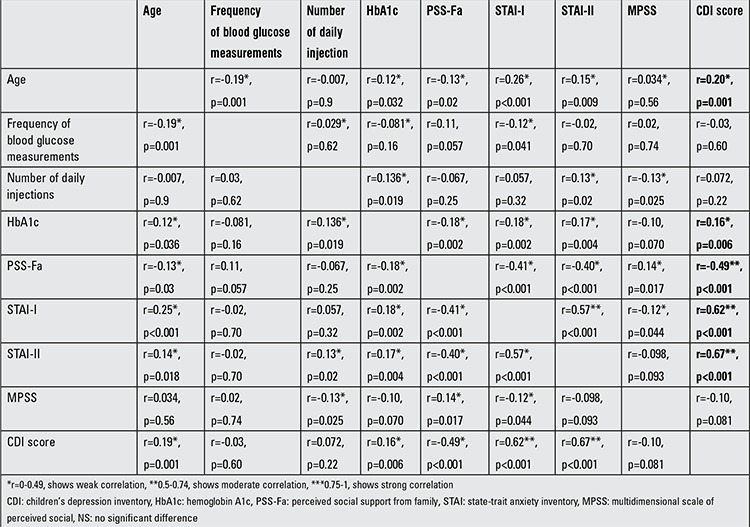
Correlation analysis between data

**Table 3:**
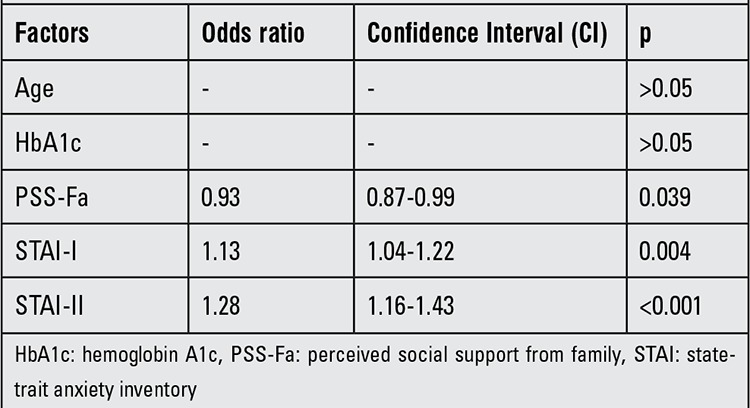
Risk factor analysis for depression
